# Exploring the landscape of tools and resources for the analysis of long non-coding RNAs

**DOI:** 10.1016/j.csbj.2023.09.041

**Published:** 2023-09-29

**Authors:** Monica Ballarino, Gerardo Pepe, Manuela Helmer-Citterich, Alessandro Palma

**Affiliations:** a Department of Biology and Biotechnologies “Charles Darwin”, Sapienza University of Rome, Piazzale Aldo Moro 5, 00161 Rome, Italy; b Department of Biology, University of Rome Tor Vergata, Via della Ricerca Scientifica, 1, 00133 Rome, Italy

**Keywords:** RNA, NcRNA, LncRNA, Bioinformatics, Transcriptomics, Genomics

## Abstract

In recent years, research on long non-coding RNAs (lncRNAs) has gained considerable attention due to the increasing number of newly identified transcripts. Several characteristics make their functional evaluation challenging, which called for the urgent need to combine molecular biology with other disciplines, including bioinformatics. Indeed, the recent development of computational pipelines and resources has greatly facilitated both the discovery and the mechanisms of action of lncRNAs. In this review, we present a curated collection of the most recent computational resources, which have been categorized into distinct groups: databases and annotation, identification and classification, interaction prediction, and structure prediction. As the repertoire of lncRNAs and their analysis tools continues to expand over the years, standardizing the computational pipelines and improving the existing annotation of lncRNAs will be crucial to facilitate functional genomics studies.

## Graphical Abstract

**Figure:**
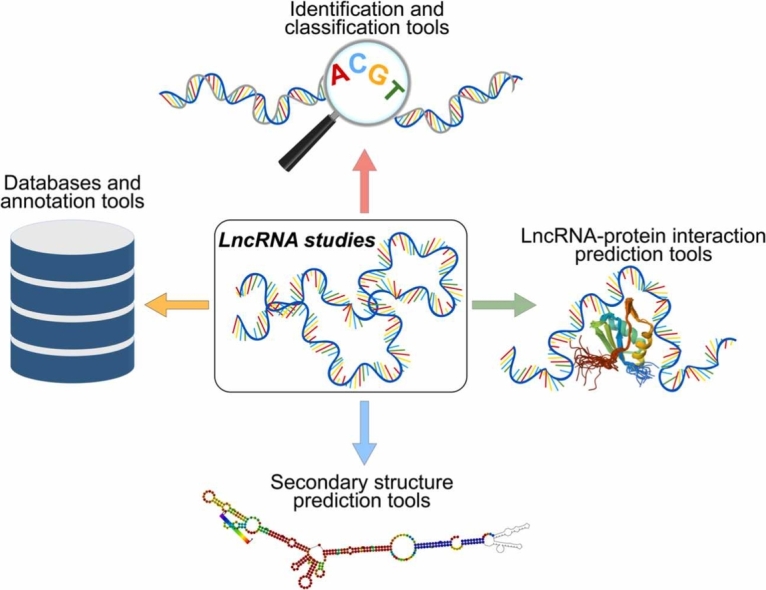

## 1 Introduction

The need to decode the complexity of living beings in terms of genetic information has recently challenged the field of molecular biology and absorbed part of the scientific interests into the study of the genome “dark side ” [1]. This called for massive efforts into the development and use of high-throughput sequencing methods culminating with the discovery of a significant fraction of non-coding (nc) genes, estimated as 17,948 according to RefSeq [2] and 19,933 in GENCODE v6 [3]. Further interpretation of these *loci* [4], [5] has led to a comprehensive mapping of their functions, which revealed the presence of a large number of regulatory elements (i.e. promoters, enhancers, silencers, insulators) and ncRNAs. More recent improvements in the high-throughput RNA sequencing technologies further expanded this collection and have put the spotlight on the class of long ncRNAs (lncRNAs). LncRNA represents a heterogeneous class of RNA-Pol II non-coding transcripts (more than 500 nt), which have attracted increasing attention of the biomedical research because of their huge assortment, number, and sequence versatility [6]. A number of genome editing approaches were applied for the generation of adequate animal model systems in which the functional significance of these RNAs was analyzed in vivo [7]. It emerged that, depending on their specific expression, subcellular distribution (nuclear and/or cytoplasmic) and interaction with other macromolecules, these RNAs can be integrated in signaling pathways controlling several physiological processes, such as cell fate, cell growth and differentiation or tissue and organ development [8]. Consequently, multiple lines of evidence link changes in lncRNA activity or abundance to various human diseases, especially cancer, cardiovascular and neurodegenerative disorders [9], [10], [11].

**Fig. 1:**
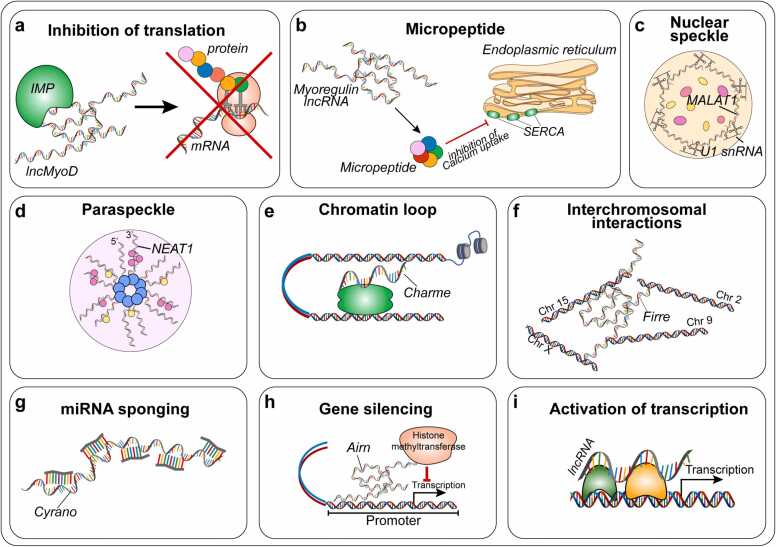
Mechanisms of action of lncRNAs. Cytoplasmic-enriched lncRNAs (a, b) can regulate mRNA translation and stability [15], [16], [19], or act as templates for micro-peptides [20], [21]. Nuclear-enriched lncRNAs (c-i) can act as enhancers [22], guides [23], decoy molecules [24], or chromatin architects [25], [26], by the interaction with a variety of transcription factors and epigenetic effectors, typically including DNA methyltransferases, histone-modifying, or chromatin remodeling enzymes.

Mechanistically, lncRNAs exert their biological functions through a wide-range of transcriptional and post-transcriptional mechanisms [12], [13] (Fig. 1). The functional plasticity of these molecules is supported by their distinctive chemical nature, enabling them to serve as scaffolds for RNA, DNA and protein partners in specific cellular compartments [14], [15], [16], [17], [18].

From these considerations, it follows that lncRNA studies can benefit the combination of molecular with genomics and transcriptomics approaches. Indeed, bioinformatics can provide a potent set of tools for lncRNA identification within the genomes of different species thus offering a new window to infer their function, in both health and disease states. Several tools have been developed to analyze massive data generated by Next Generation Sequencing (NGS) technologies for the *de novo* discovery of lncRNAs; other computational strategies can help in assessing the genomic location, predicting the structure, thus aiding the interpretation of the potential lncRNA functions. Furthermore, other *in silico* tools able to predict the interactions between lncRNAs and other biomolecules can be widely used to provide insights into the study of lncRNA-mediated biological networks. Finally, integrating prior knowledge with lncRNA expression data and identifying differentially expressed lncRNAs that participate in specific cellular pathways are useful approaches for studying these molecules effectively.

In this review, we summarize the collection of computational resources available for facilitating lncRNA research, classifying them into several categories to support annotation, classification, RNA/protein interactions, and structure analyses. Some of these tools have multiple features or functions and will be classified according to their primary use.

## 2 Results

### 2.1 Databases and annotation

Public databases are pivotal sources of scientific content. Annotation tools and resources offer the possibility to classify, standardize nomenclature by cross-referencing other databases and retrieve functional information about any given manually or computationally curated biological entities. In the last decade, many databases have been implemented with multiple ncRNA notions, such as sequences, interactions, and gene ontologies. In this direction, a huge international effort recently led to build RNAcentral [27], a comprehensive aggregator database aiming to provide a reference resource of sequences and annotations for researchers studying ncRNAs. Currently, the database stores over 35 million unique sequences from over 170,000 organisms and it contains annotations for almost all types of ncRNAs, including transfer RNAs (tRNAs), ribosomal RNAs (rRNAs), small nucleolar RNAs (snoRNAs), microRNAs (miRNAs) and lncRNAs. In RNAcentral, annotations are provided by expert curators and extracted from a variety of sources, including literature and experimental data, with cross-references to other resources. Importantly, a total of 51 databases are imported into RNAcentral, including some that are completely dedicated to lncRNA information, such as EVLncRNAs [28], LncBase [29], LncBook [30], LNCipedia [31] and lncRNAdb [32]. Users can mine RNAcentral by sequence, identifier, or keyword and can access a variety of tools for analyzing and visualizing data. RNAcentral also provides web services and application programming interfaces (APIs) that allow integration with other *in silico* resources. Among the RNAcentral hosted databases, LncBook and LNCipedia represent invaluable tools for lncRNA annotation [30], [31]. Specifically, LncBook stores 95,243 human lncRNA genes, which are integrated with annotations at different omic levels, such as their conservation across species, nucleotide variations, methylation, expression and interactions with miRNA and proteins. Other tools allow the users to perform identifier conversion across different databases, compute the coding potential and check the genomic location of lncRNAs. LNCipedia cures the collection of 56,946 lncRNA genes and facilitates both literature search and the download of tracks for genome browsers, such as IGV and UCSC.

The functional importance of lncRNA-mediated networks and interactions has recently led systems biology to develop tools for data storing and integration. In this direction, the database RAIN (RNA-protein Association and Interaction Networks) [33] stores lncRNA-RNA and lncRNA-protein interaction data integrated with the protein-protein interaction STRING database [34], thus facilitating the exploration of regulatory networks involving lncRNAs. Similarly, ncFANs [35] is a web-based resource for functional annotation of lncRNAs that implements three distinct functional modules, for i) retrieving ncRNAs-protein coding genes relations, ii) identifying enhancer-derived lncRNAs, and iii) performing functional annotation through microarray-based analysis. Users are allowed to access information related to the expression of annotated lncRNAs and interaction networks in both physiological and pathological contexts, with a particular emphasis on cancer.

Other freely available databases include NPInter [36], which provides comprehensive annotations of lncRNA-protein interactions (i.e. binding affinity, localization, and function) in multiple species, including human, mice, and rats. It also includes lncRNA-DNA interactions obtained through the Chromatin Isolation by RNA Purification (ChIRP-seq) technique, as well as interactions involving circular RNAs. Furthermore, disease associations have been incorporated into the database. Another resource that integrates experimentally validated and computationally predicted RNA interactions from literature mining and databases is RNAInter4.0 [37]. RNAInter4.0 provides information about various types of interactions across 8 different *taxa*, including RNA-RNA, RNA-protein, RNA-DNA, RNA-compound, and RNA-histone modification interactions.

Furthermore, RNA-Chrom [38] is a recently established database that provides manually curated information on RNA-chromatin interactions. This valuable resource contains the coordinates of billions of chromatin interactions involving thousands of RNAs from human and mouse. Despite the progress made so far, novel experimental methods for identifying lncRNA interactions continue to be time-consuming and costly. Manually annotated databases, therefore, promote the development of computational approaches that serve as a complementary strategy to facilitate experimental work [39]. Importantly, annotation of any genetic sequences, including lncRNAs, can be found in the NCBI GeneBank database [40], a publicly accessible repository containing nucleotide sequences, as well as gene expression data and genetic variation data from a variety of organisms, including viruses, bacteria, fungi, plants, and animals.

Some of the available resources let users also explore RNA-RNA interactions, thus helping in the relations between lncRNAs and other types of short and long RNAs. An example is RISE (RNA Interactome from Sequencing Experiments) [41], a repository of 328,811 RNA-RNA interactions built from experimental (transcriptome-wide and targeted studies) and *in silico* data taken from other sources, such as NPInter [36], RAIN [33] and RAID [42] databases. RISE includes data from human, mouse, and yeast, and provides a web interface with a search box in which users are allowed to retrieve information on RNA-RNA interactions for a specific species in both graph and tabular forms. LncRRIsearch [43] is a web server in which users can input a query and a target RNA by its gene/transcript name or ID and choose between human or mouse species. Finally, the web server TANRIC (The Atlas of non-coding RNA in Cancer) [44] integrates gene expression data from multiple cancer types, mostly by the Cancer Genome Atlas project to explore the correlation between lncRNA expression and clinical metadata, within and across the different tumor types.

Growing evidence suggests that subcellular localization of lncRNAs could offer insights into their functionality. On this direction, in 2018 the database lncSLdb [45] was introduced with the aim of enhancing our understanding of the subcellular localization of lncRNAs. This resource was established to store and effectively manage qualitative and quantitative subcellular localization data of lncRNAs obtained through literature mining, thereby contributing to the expansion of knowledge in this field. Here, the authors have classified the transcripts into three fundamental localization types (nucleus, cytoplasm, and nucleus/cytoplasm) based on the accumulated regions of lncRNAs. Because of the scarcity of experimental data, various algorithms have also been developed to predict and annotate the subcellular localization of lncRNAs, including lncLocation [46], GM-lncLoc [47], and GraphLncLoc [48]. In perspective, standardization of both annotation and nomenclature, will improve the lncRNA knowledge and will advantage the integration of large volumes of lncRNA data from different sources. Using multiple tools and resources in combination could enhance the accuracy of the retrieved information.

### 2.2 Identification, classification and annotation

*De novo* identification of lncRNAs poses a challenging and non-trivial task which often requires a combination of both experimental and computational methodologies. One popular approach involves utilizing transcriptome assembly techniques that rely on high-throughput RNA sequencing (RNA-seq) data. These methodologies rely on *de novo* assembly algorithms, such as Trinity [49], Oases [50] or SPAdes [51]. To identify *bona fide* non-coding RNAs, the assembled transcripts can be further filtered based on other specific criteria, such as transcript length, exon-intron structure, and protein-coding potential [52], often using and combining two or more different tools.

The tools available for lncRNA annotation and coding-potential assessment, which are currently operational at the time of manuscript writing, are enlisted in Table 1.

**Table 1:** Tools and resources for *de novo* annotation and functional analysis of lncRNAs.

**Tool name**	**Acronym**	**Model/Method**	**Web link**	**Reference**	**notes**
Coding Potential Assessment Tool	CPAT	Logistic regression	https://code.google.com/archive/p/cpat/ https://rna-cpat.sourceforge.net/	[53]	It requires programming skills. BED or FASTA files required as input
FlExible Extraction of LncRNAs	FEELnc	Random Forest	https://github.com/tderrien/FEELnc	[54]	It requires programming skills. GTF or FASTA files required as input
Predictor of long non-coding RNAs and messenger RNAs based on an improved k-mer scheme	PLEK	k-mer and Support Vector Machine	http://202.200.112.245/plek/	[55]	It requires programming skills. FASTA file required as input
Coding Potential Calculator, lncRNA Orthologs and Multiple Evidence	COME	-	https://github.com/lulab/COME	[56]	It requires programming skills. GTF file required as input
EVlncRNA-pred	-	Multilayer Neural Network	http://biophy.dzu.edu.cn/lncrnapred/index.html	[57]	No programming skills are required. A GTF annotation file is required as input.
lncRScan-SVM	-	Support Vector Machine	-	[58]	It requires programming skills. GTF and FASTA files required as input
Phylogenetic Codon Substitution Frequencies	PhyloCSF	Phylogenetic codon substitution frequency	https://data.broadinstitute.org/compbio1/PhyloCSFtracks/trackHub/hub.DOC.html https://github.com/mlin/PhyloCSF/wiki	[59]	It requires programming skills. Scores for selected phylogenies may be displayed with the UCSC genome browser
RNAcode	-	Support Vector Machine	https://github.com/ViennaRNA/RNAcode	[60]	Provided as both software and web services
LnCompare	LnCompare	-	http://www.rnanut.net/lncompare/	[61]	It allows the functional comparison between two sets of lncRNA
LncSEA	LncSEA	-	https://bio.liclab.net/LncSEA/	[62]	Gene set and functional enrichment analysis on lncRNAs

Most of the available tools for the assessment of the coding potential of lncRNAs utilize primary sequence and/or structural information. For instance, CPAT (Coding Potential Assessment Tool)[53], is a machine learning-based tool that leverages sequence features to distinguish between coding and non-coding RNAs. CPAT assists the discovery of lncRNA from transcriptomic data employing a logistic regression model in a selected list of organisms by providing sequences in FASTA or BED formats. Other tools employ alignment-free methodologies and integrate several molecular features. One example is FEELnc (FlExible Extraction of LncRNAs)[54], a tool for lncRNA annotation which classifies transcripts as protein-coding or non-coding through a Random Forest model. FEELnc integrates multiple features, including sequence conservation, secondary structure, and the length of potential open reading frames (ORF). Another alignment-free tool is PLEK (predictor of long non-coding RNAs and messenger RNAs based on an improved k-mer scheme) [55]. PLEK is an open-source computational resource that uses a k-mer scheme and a support vector machine (SVM) algorithm to identify lncRNAs in the absence of genomic sequences or annotations. Li and co-authors recommend its preferable use with PacBio or 454 sequencing data and large-scale transcriptome data. Another example is COME (coding potential calculator based on multiple features) [56], which is based on the observation that lncRNAs generally lack coding potential and do not have significant sequence similarity with protein-coding genes. This resource applies a supervised model to identify lncRNAs from sequence features and experimental evidence by using a decompose-compose method.

Other tools utilize RNAseq data for lncRNA identification. Among them lncEvo [63] is a tool for the identification and conservation of lncRNAs which uses a workflow made of three major tasks: transcriptome assembly from RNAseq data, prediction of lncRNA, and genome-wide analysis of lncRNA conservation between two species.

EVlncRNA-pred [57] is a three-layered deep-learning neural network-based tool that distinguishes lncRNAs validated by high- from those derived from low-throughput experiments often causing sequencing noise, excluding coding transcripts. A specific module of this algorithm, named EVlncRNA-Dpred, is also available as a webserver, and uses a GTF annotation file as input. LncRScan-SVM [58] is a machine learning-based approach that uses a SVM algorithm to predict whether a transcript is protein-coding or not. By using a combination of gene structure, transcript sequence, potential codon sequence and conservation, LncRScan-SVM produces a score which is used for calculation of coding/non-coding potential. A different approach is undertaken by PhyloCSF (Phylogenetic Codon Substitution Frequencies) [59], a comparative genomics-based tool that predicts the coding potential based on evolutionary conservation. To distinguish between coding and non-coding sequences, PhyloCSF uses two phylogenetic models, one for predicting the evolution of codons into coding genetic material, and the other for the evolution of codons into non-coding genes.

RNAcode [60] is a program aimed at detecting coding regions in multiple sequence alignments. Differently from other methods, it does not rely on the use of any machine learning components, as it is based on universal evolutionary signatures of coding sequence.

Collectively, these tools can identify lncRNAs by incorporating diverse features, such as primary sequences of RNAs and other information, including multiple sequence alignments. Frequently, a machine learning methodology underpins each of these tools to facilitate the training and construction of a model, which can accurately infer the coding potential. Many of these methods have been extensively compared [64] as a guide to their use. In fact, as these tools can be sensitive to the quality of transcriptome assembly or to the intrinsic features of the targeted RNAs, their choice must rely on the specific research purposes and data type. The combination of multiple tools is also beneficial.

LncRNA genes can also be identified by other functional characteristics, such as the presence of neighboring transcription factor binding sites or their proximity to specific chromatin domains.

Several web servers and tools have been developed to help in deciphering the functions of lncRNAs. These tools, which include Co-LncRNA [65], Lnc-GFP [66], and FARNA [67], can be used to predict the function of selected lncRNAs using RNA-seq data and to examine their expression correlation with mRNAs. LnCompare [61] provides the opportunity to analyze lncRNA set features through distinct modules: one for comparing two sets of lncRNAs to identify significantly different features; the other for retrieving a set of lncRNAs that are similar to user-defined query genes. LncSEA [62] integrates various available resources of human lncRNAs to allow users to perform annotation and enrichment analyses on the submitted lncRNA lists. In its latest version, LncSEA provides support for over 400,000 reference sets, which have been categorized (n = 33) into downstream (e.g., chromatin, RNA or protein interactions, eQTLs) or upstream regulators of the lncRNA functions thanks to the integration of TF-ChIP-seq, DNase-seq, ATAC-seq and H3K27ac-ChIP-seq data. Results from gene set enrichment analyses are provided within a web interface, requiring a list of lncRNAs and the adjustment of a few user-defined statistical parameters.

Overall, various bioinformatics and biostatistics methodologies can be employed in conjunction with experimental approaches, even if an exploration of these methodologies is beyond the scope of this review. However, experimental approaches aimed at identifying lncRNAs at both genome and transcriptome levels could be needed for a definitive identification of lncRNAs and their and annotation on public repositories.

### 2.3 Predicting interactions of lncRNAs with proteins or nucleic acids

LncRNAs regulate gene expression through interactions with other molecules. For instance, they can engage with proteins in several ways, including direct binding, recruiting proteins to specific genomic *loci*, and regulating protein function, in both the nuclear and cytoplasmic compartments [6]. Therefore, predicting the protein partners of lncRNAs is vital for understanding their role in any given biological and molecular context.

Henceforth, we list some available bioinformatic tools for predicting lncRNA-protein interactions (Table 2), which are also reviewed in [68]. Most of the available methods are based on the analysis of the primary sequence of either the RNA or the protein provided as inputs. Some of them also consider some structural features or differ in the machine learning algorithm leveraged for training the model and making predictions.

**Table 2:** Tools and resources for the prediction of protein and nucleic acids lncRNA-interactions.

**Name**	**Acronym**	**Model/Method**	**Web-link**	**Reference**	**notes**
RNA-protein interactions using only Sequence information	RPISeq	SVM or RF	http://pridb.gdcb.iastate.edu/RPISeq/	[69]	It requires protein and RNA sequences in plain text format as input
RPITER	RPITER	CNN with stacked auto-encoder	https://github.com/Pengeace/RPITER	[70]	It requires python language programming
Deep Mining ncRNA-Protein Interactions	DM-RPIs	SVM, RF, CNN with Deep Stacking Auto-encoders Networks	-	[71]	Methods provided within the article
Ensemble deep learning framework with multi-scale features combination	EDLMFC	Ensemble deep learning with CNN and bi-directional long short-term memory network (BLSTM)	https://github.com/JingjingWang-87/EDLMFC	[72]	It requires python language programming
Interaction Pattern Miner	IPMiner	stacked autoencoder, RF	https://github.com/xypan1232/IPMiner	[73]	It requires python language programming
Prediction of lncRNA-Protein Interactions using HeteSim Scores	PLPIHS	HeteSim Scores and SVM	-	[74]	Methods provided within the article
HLPI-Ensemble	-	SVM, RF, XGB	http://112.126.70.33/hlpiensemble/prediction.php	[75]	It requires protein and RNA sequences in plain text format as input
RPI-SE	RPI-SE	Stacked ensemble	https://github.com/haichengyi/RPI-SE	[76]	It requires python language programming
Predicting Long Non-Coding RNA and Protein Interaction Using Graph Regularized Nonnegative Matrix Factorization	LPGNMF	graph regularized nonnegative matrix factorization (LPGNMF)	-	[77]	Methods provided within the article
BGFE	BGFE	RF, stacked auto-encoder network	-	[78]	Methods provided within the article
*cat*RAPID	*cat*RAPID *signature/omics*	Methods provided within the articles and website	http://s.tartaglialab.com/page/catrapid_group	[79], [80]	It requires protein and RNA sequences in plain text format as input and the setting of user-defined parameters
RBPsuite	RBPsuite	iDeepS (CNNs and LSTMs), and CRIP (stacked codon-based encoding scheme, CNN and a biLSTM)	http://www.csbio.sjtu.edu.cn/bioinf/RBPsuite/	[81]	It requires RNA sequence in plain text format as input and the setting of user-defined parameters
omiXcore	omiXcore	*-*	http://service.tartaglialab.com/update_submission/742489/8e5af8ea58	[82]	Web server. It requires protein and RNA sequences in plain text format as input. No programming skills required
Sequence and structure motif enrichment analysis for ranked RNA data from in vivo binding experiments	SMARTIV	*-*	http://smartiv.technion.ac.il/	[83]	Web server. It requires RNA sequence in BED or FASTA formats.
Protein-RNA Interaction by Structure-informed Modeling using deep neural NETwork	PrismNet	Software and architecture provided within the original article	https://github.com/kuixu/PrismNet	[84]	It requires programming language skills
LncADeep	LncADeep	deep belief network (DBN) for lncRNAs identification, and deep neural networks for lncRNAs functional annotation	https://github.com/cyang235/LncADeep	[85]	It requires programming language skills. Files in FASTA format required for both lncRNAs identification and annotation
miRanda	miRanda	Smith-Waterman-like algorithm	https://bioweb.pasteur.fr/packages/pack@miRanda@3.3a	[86]	Programming skills required.
TargetScan	TargetScan	CNN	https://www.targetscan.org/vert_80/	[87]	Web server. It requires genes or miRNA identifiers as input, and setting user-defined parameters
Mienturnet	Mienturnet	over-representation of miRNA-target interactions (from TargetScan and miRTarBase data)	http://userver.bio.uniroma1.it/apps/mienturnet/	[88]	Web server. It requires gene or miRNA identifiers as input.

RPISeq [69] is an example of computational tool that predicts RNA-protein interactions by using sequence-derived information. The method generates a set of features from the RNA and protein sequences, which are then used to train two classifiers, a SVM and a Random Forest (RF), on a set of known RNA-protein interactions. Once trained, the classifier can be used to predict the likelihood of RNA and protein interactions between any sequences, regardless of the organism of origin. RPITER [70] is a hierarchical deep learning-based framework which feeds an algorithm consisting of four ensemble-integrated basic modules with the RNA and protein sequences as input.

BGFE [78] is a sequence-based method that uses a Stacked auto-encoder network together with a RF classifier as model. The model is primarily fed with ncRNA sequences that are represented by a k-mers sparse matrix, then a singular value decomposition (SVD) is used to extract feature vectors from this matrix. Evolutionary information is extracted from protein sequences through a PSSM, and a bi-gram algorithm used to extract feature vectors from the matrices. Finally, a RF classifier is fed with the data to predict the putative ncRNA-protein interaction.

RBPsuite [81] is a webserver designed to predict RNA-binding protein (RBP) binding sites on both linear and circular RNAs using a deep learning approach. Non-deep-learning-based tools include omiXcore [82] and SMARTIV [83]. OmiXcore is an RBP-general method, which employs a non-linear algorithm on pooled RNA-protein interactions, accepting protein and large RNA sequences as input. SMARTIV requires a set of RNA sequences in BED format and utilizes Hidden Markov Model (HMM) to find the enriched sequence and structural motifs from in vivo binding data.

DM-RPIs (Deep Mining ncRNA-Protein Interactions) [71] uses RNA and protein sequences as input to predict the probability of their interaction. The model is based on three machine learning classifiers, namely SVM, RF, and Convolutional Neural Network (CNN), which are separately trained as individual predictors and then integrated using a stacked ensemble strategy. EDLMFC (Ensemble deep learning framework with multi-scale features combination) [72] is a computational methodology predicting ncRNA-protein interactions by combination of multiple features, including primary sequences or RNA and protein structures. These features are learned by layered networks, including CNN and “Bidirectional Long Short-Term Memory” (BLSTM).

IPMiner (Interaction Pattern Miner) [73] is a tool based on deep learning and stacked ensembling with a reported high prediction performance achieved by integrating different predictors. Instead, PLPIHS (Prediction of lncRNA-Protein Interactions using HeteSim Scores) [74] improves the accuracy of predictions by utilizing a learning framework combined with HeteSim measurements. Specifically, the model first builds a heterogeneous network based on lncRNA-lncRNA similarity, lncRNA-protein association, and protein-protein interaction networks. Then, PLPIHS calculates the similarity score using the HeteSim metric for each pair of lncRNA-protein associations, under each path. Finally, an SVM classifier is built with the HeteSim scores to predict lncRNA-protein interactions.

HLPI-Ensemble [75] is a method designed specifically for human lncRNA-protein interactions, onto which the model is trained. HLPI-Ensemble adopts an ensemble strategy based on the combination of three different machine learning algorithms: SVM, RF, and Extreme Gradient Boosting (XGB). RPI-SE [76] upgrades the previous RPI-SAN by integrating the Gradient Boosting Decision Tree, SVM and Extremely Randomized Trees (ExtraTree) algorithms. Position weighted matrix and k-mer sparse matrix first mine features from protein and RNA sequences, then a stacking ensemble approach is used to integrate the predictors.

LPGNMF (Predicting Long Non-Coding RNA and Protein Interaction Using Graph Regularized Nonnegative Matrix Factorization) [77], is a tool designed to capture complex relationships between lncRNAs and proteins. Unlike other tools, it uses as input the quantitative expression levels of lncRNAs and proteins in in their respective biological context. The obtained matrix is factorized into two non-negative matrices, representing the latent features of lncRNAs and proteins, respectively. These features are subsequently used to calculate the “similarity score”, as the likelihood of interaction of a given lncRNA-protein pair.

catRAPID [89] computes protein-RNA interaction propensities taking into consideration not only primary (RNA and protein) sequence information but also other biochemical features, including secondary structures, hydrogen bonding, and van der Waals forces. Users are allowed to choose a specific implementation to reconstruct the interaction score for protein-RNA pairs or rank the fragments of long protein and RNA sequences according to the predicted interaction strength. It has been recently upgraded to catRAPID omics 2.0 [80], which is a webserver that allows users to input protein or RNA sequences to calculate the interaction scores. In the last upgrade, it is also possible to predict the interactions between a custom protein set and a custom RNA set, and to display the predicted binding sites for both protein and RNA sequences.

PrismNet (Protein-RNA Interaction by Structure-informed Modeling using deep neural NETwork) [84] is another deep learning-based tool that integrates RNA structure data and RBP binding data to predict RBP binding sites at the nucleotide level.

LncADeep [85] is a tool for both the annotation and the prediction of lncRNA interactions with proteins. It is based on a deep neural network architecture and takes, as input, the sequences of the lncRNA and protein molecules, as well as their predicted secondary structures. It uses a neural network model trained on a dataset of known lncRNA-protein interactions. Once the model is trained, it can be used to predict the likelihood of interaction between any given lncRNA and protein.

One key aspect of lncRNAs relies on their ability to functionally bind not only to proteins but also to nucleic acid sequences, including other RNA molecules. This binding capacity is reflected in their functioning as post-transcriptional regulators, being able to influence the expression of distinct genes directly or indirectly. Notoriously, lncRNAs can function as sponges for miRNAs [90], [91], [92], [93], hence regulating the expression of target genes at a post-transcriptional level. Different tools have been developed to infer miRNA binding sites on nucleic acid sequences, such as miRanda [86], TargetScan [87] and Mienturnet [88]. MiRanda is an algorithm for the prediction on miRNA binding sites on genomic sequences based on sequence complementarity and the thermodynamic stability of RNA duplexes. TargetScan is a tool for the prediction of miRNA target sites that are conserved in 3′ UTRs, also offering customized methods for ranking the predictions. Mienturnet is a web- and R-based tool that enables the discovery of miRNA binding sites on RNAs. Given a list of miRNAs or genes, it can output computationally predicted or experimentally validated miRNA-target interactions.

In line with this, the output may serve as a starting point for conducting further experimental validations, such as performing cross-linking immunoprecipitation (CLIP) or reciprocal RNA pull-down approaches [94], [95].

All these tools enable the prediction of putative interactions between lncRNAs and proteins or nucleic acids. Since the accuracy of these tools can vary depending on several aspects, including the datasets used for training, it is advisable to proceed with caution when inferring interactions with other molecular entities.

### 2.4 Structure prediction and comparison

The formation of RNA secondary structures has been shown to drive the scaffolding activities of lncRNAs [18], [96]. In fact, the spatial arrangement achieved through dynamic base-pairing interactions can facilitate interactions of lncRNAs with distinct molecules, thus forming proper ribonucleoprotein hubs for the downstream modulation of gene expression. Secondary structures are also important for their localization within the cell, and for the stability of the lncRNA in its cellular context [97], [98].

Although necessary, experimental procedures aimed at assessing the RNAs secondary structure can be time-consuming and expensive, leading to an increasing demand for automated tools to facilitate structure prediction. Considering this need, a plethora of algorithms and online resources have emerged in recent years, and can be used for studying RNA, and more specifically, lncRNAs (Table 3).

**Table 3:** Tools and resources for lncRNA secondary structure prediction and comparison.

**Name**	**Tool type**	**Notes**	**Web-link**	**Reference**
ViennaRNA Web Services	Web server	Also available as command line tools	http://rna.tbi.univie.ac.at/	[99]
RNAstructure	Web server	Features available: download of RNA structures	https://www.urmc.rochester.edu/rna/	[100]
PknotsRG	Web server	For pseudoknock structures	http://bibiserv.techfak.uni-bielefeld.de/pknotsrg	[101]
Iterative HFold	Command line tool		https://github.com/HosnaJabbari/Iterative-HFold	[102]
Rtools	Web server		http://rtools.cbrc.jp/	[103], [104]
Knotify+	Command line tool		https://github.com/ntua-dslab/knotify	[105]
Rtips	Web server	It includes IPknot+ + and RactIP	http://ws.sato-lab.org/rtips/	[106], [107], [108]
Web-Beagle	Web server	It requires RNA sequence in plain text format as input and the setting of a few parameters	http://beagle.bio.uniroma2.it/	[109]
MultiSETTER	Web server	It requires programming skills	http://siret.ms.mff.cuni.cz/multisetter-app	[110]
BRIO	Web server	It requires RNA sequence in plain text and/or dot-bracket annotation of RNA sequence as input(s)	http://brio.bio.uniroma2.it/	[111]

One of the most popular resources is ViennaRNA Web Services [99], a server providing a suite of online tools, including some for RNA secondary structure prediction and analysis. The resource is based on the ViennaRNA package, a set of programs dedicated to RNA secondary structure prediction, RNA folding kinetics, and RNA-RNA interaction prediction. ViennaRNA offers several online tools, including RNAfold, a popular tool for predicting the secondary structure of RNAs using the minimum free energy (MFE) method to predict the most stable secondary structure for a given RNA sequence. RNAstructure [100] represents another option and a web server has been developed to lend accessibility to non-expert users.

One major problem in predicting the secondary structure of lncRNAs is the existence of pseudoknots, which are complex secondary structures that arise when a single-stranded loop in the RNA base-pairs with another RNA region which is not adjacent in the primary sequence. The result is a structure in which two or more stem-loop structures are interlinked and create a “knot-like” appearance. PknotsRG [101], IPknot [112], Iterative HFold [102] and Rtools [104], incorporate specific algorithms to predict pseudoknot structures. In addition, Rtips (RNA sTructure prediction using IP Scheme) [106] is a web server in which IPknot is combined with RactIP [106], the latter for predicting RNA-RNA interactions with kissing hairpins. Other methodologies, like Knotify+ [105], address pseudoknots prediction by taking advantage of the combination between context-free grammar, maximum base pairing, and minimum free energy.

The existence of tools that allow the comparison between RNA secondary structures is advantageous for researchers aiming to understand and interpret the relationships between different RNAs as well as differences and similarities among secondary structures inside the same RNA molecule. Web servers for the comparison of RNA sequences and secondary structures include Web-Beagle [109] and MultiSETTER [110]. Web-Beagle performs RNA structural alignments taking sets of RNA sequences and structures or primary sequences alone as input. In the absence of known secondary structures, the server makes predictions by using the RNAfold algorithm. Specifically, it performs structural comparisons between secondary structures, generating pairwise alignments, assessing structural similarity, and evaluating statistical significance for each alignment. This resource can also be used for the identification of homologous regions shared by different RNAs, or for functional annotation. MultiSETTER is a web server for the analysis and visualization of RNA structure in the space, based on an algorithm that performs the alignment of multiple RNA structures. The inputs can be either a list of Protein Data Bank (PDB) IDs, or user-defined text files. The algorithm outputs the three-dimensional structure of the RNAs together with reports and statistics.

The identification of sequences or structure motifs in the RNA serves as a fundamental step in the discovery of potential RNA interactors. From this perspective, Adinolfi and collaborators [113] shed light on intriguing motifs that exhibit a higher occurrence in mRNAs targeted by specific ncRNAs, particularly lncRNAs. The dataset comprises 2508 sequence and 2296 structure motifs, which are associated with the binding of 186 individual proteins and 69 single protein domains. Based on this dataset, Guarracino and collaborators [111] have developed BRIO, a web server designed to identify sequences or structural motifs potentially involved in the interaction between lncRNAs and RNA-binding proteins. The database contains more than 2000 RNA motifs that are known to bind human proteins from PAR-CLIP, eCLIP, HITS experiments. Using a substitution matrix, it returns the list of protein binding motifs identified in the input sequences.

In summary, several tools and web services exist for the prediction and comparison of lncRNA secondary structures. These tools use various approaches, including MFE methods, pseudoknot prediction, and deep learning for their predictions, providing advantages for functional studies on lncRNAs. Care should be taken when using computational tools to infer lncRNA functions on secondary structure predictions. Recent papers have highlighted some pitfalls in statistical methodologies behind those predictions, including the use of comparative sequence analysis [114], [115].

## 3 Relevant considerations in the study of lncRNAs

Over the past few years, increasing attention was given to link lncRNA expression and nucleotide variations in genetic and complex diseases. Although next-generation RNA sequencing approaches have revealed numerous alterations in their expression, the implication of lncRNAs in disease is still in its infancy and demands further annotation and targeted methodologies. The improvement of deep-sequencing technologies made possible to acquire lncRNA sequences and study their mutations in cancer-related processes. These alterations include single nucleotide variations, indels, and copy number amplifications affecting non-coding regions of the genome [116]. Some lncRNAs, such as the well-characterized H19, NORAT, MALAT1 and HOTAIR, have been also implicated in promoting cancer metastasis, via mechanisms that include epithelial-mesenchymal transition, migration and modulation of the microenvironment [117], eventually affecting cancer-associated signaling pathways [118]. Moreover, it has been shown that lncRNAs can function either as tumor-suppressors or have an oncogenic function [119]. Based on these considerations, it is advisable that dedicated tools and resources are necessary to enhance our understanding of the implication of lncRNAs in cancer. Recently, significant efforts have been made to store and annotate lncRNAs with validated cancer roles. This has resulted in the creation of a resource called the “Cancer lncRNA Census” [120], which stores 122 lncRNAs from GENCODE with an established role in cancer phenotypes. An additional resource is lncRNAfunc [121], a knowledgebase of human lncRNAs with roles in cancer. It integrates data from various tumor types from The Cancer Genome to gain insights into pathological mechanisms mediated by lncRNAs.

LncRNAs have also been associated with diseases other than cancer, as a consequence of mutations occurring in their sequence and regulatory regions. Many studies have linked lncRNAs to a broad spectrum of diseases, including cardiometabolic traits [122], autism [123], amyotrophic lateral sclerosis [124], among others. In line with this, a resource named LncRNADisease [125] has been developed as a compendium of experimentally validated and predicted ncRNA-disease associations derived from manual curation of literature and other resources.

The discovery of disease-associated lncRNAs, which could act through either direct or indirect pathogenic mechanisms, has been enhanced by the increasing use of research methodologies aimed at identifying genomic variants associated with diseases or traits, such as GWAS studies. In this context, a recent work led to the development of lncRNASNP [126], a repository of single nucleotide polymorphisms (SNPs) located within lncRNA sequences, along with their consequences on the molecular structure and function of these lncRNAs. The resource also includes drug target associations, GWAS, and the effect of SNPs on expression quantitative trait *loci* (eQTL).

## 4 Conclusions and future directions

The availability of computational tools capable of predicting the structural attributes, functional characteristics, and intermolecular interactions of RNA represents a valuable source for a more comprehensive understanding of the “dark side” of the genome. In the case of lncRNAs, the application of specialized bioinformatic pipelines, exclusively designed for analyzing this class of transcripts, is helping scientists to bridge the gap between the existence of still poorly characterized non-coding sequences and their possible impact on gene regulation.

A plethora of bioinformatics tools has been developed to analyze the expression and regulation of lncRNAs. Broadly employed for the analysis of RNA sequencing data, prominent tools include DESeq2 [127], edgeR [128], and Limma [129]. In fact, differentially expressed genes derived from these analyses often include lncRNAs. However, a critical obstacle persists in the form of limited lncRNA annotation data in databases governing both pathways and gene ontology enrichments, which impedes accurate assessment of their involvement in distinct pathways or cellular processes.

Scientists are also aware that predictive tools would require extra validations by means of experimental approaches. In fact, the advance of *in silico* tools, that are thought for their direct and immediate application, is accompanied by the need of new structural, molecular and biochemical bench-based approaches. These can be either RNA or protein-centric methods for the analysis of RNA-protein interactions [130], or refined with the use of psoralen or dextran sulfate for improving the study of RNA-RNA interactions [131], [132]. These biochemical approaches are increasing the stringency and the accuracy to assess in vivo the predictions obtained using bioinformatics pipelines.

One of the areas that will undoubtedly require time and resources in the coming years is the standardization of the nomenclature for lncRNAs. The lack of standardized nomenclature for lncRNAs can lead to confusion and hinder data sharing and collaboration. Establishing clear and consistent naming conventions is crucial to facilitate communication and ensure that findings can be effectively integrated into the broader scientific community. Another crucial aspect is related to understanding the tissue-specific and condition-specific roles of lncRNAs, which is vital for unraveling their functions in health and disease. However, obtaining relevant data can be challenging, especially for rare cell types or under specific conditions. Furthermore, considering that lncRNAs often exhibit functional redundancy, where multiple lncRNAs may regulate the same genes or pathways, deciphering the individual contributions of these lncRNAs to cellular processes and disease states can be complex and requires sophisticated experimental design and analysis.

Interdisciplinary collaboration from molecular biology, bioinformatics and related disciplines is expected to increase to make scientists more trustworthy and to address various issues related to the study of lncRNAs. This will strength open communication and more critical discussion on the importance of wet-lab approaches, such as CLIP-seq and PAR-CLIP-seq for the analysis of lncRNAs interactions. Training on large datasets and experimental validation will also beneficial for implementation of more accurate computational predictors and for the use of NGS methodologies to comprehensively profiling lncRNAs.

## CRediT authorship contribution statement

Conceptualization: A.P., M.B. Data curation, formal analysis, investigation and methodology: A.P., M.B., G.P., M.H.C. Writing – review & editing: A.P., M.B., G.P., M.H.C. Project administration: A.P., M.B. Funding acquisition. M.B. All authors have commented on and approved the final version of the manuscript.

## Declaration of Competing Interest

The authors declare no competing interest.
